# Rocketship and the Rural Health Workforce Revolution in the Pacific: Growing Skilled Medical Generalists Across the “Blue Continent”

**DOI:** 10.3389/fpubh.2020.612531

**Published:** 2021-02-03

**Authors:** Lachlan McIver, Dan Manahan, Sam Jones, Lisiate 'Ulufonua

**Affiliations:** ^1^Rocketship Pacific Ltd., Melbourne, VIC, Australia; ^2^Ministry of Health, Tongatapu, Tonga

**Keywords:** rural, medical, Pacific, workforce, training, island, generalist

## Abstract

Dramatic shifts are occurring in the size, shape and skill of rural health workforces in Pacific island countries (PICs) due to an unprecedented convergence of political agreement, policy commitment, donor support and technical assistance. In particular, the impact of “medical internationalism” is being felt across the Pacific region, with new doctors returning home in far greater numbers than ever before, the majority having graduated from medical schools in Cuba, China and other countries outside the region, in addition to the more typical numbers graduating and returning home from the region's main medical schools in Fiji and Papua New Guinea. With an agreed regional vision of “Healthy Islands” across the Pacific, the main objective of expanding overseas training opportunities for Pacific island medical students has been to correct the widespread centralization and maldistribution of the medical workforce in PICs and improve health access and quality of care in rural areas by deploying the new graduates to outer-island facilities. However, the return of these new graduates in several PICs has demonstrated that additional training is required to equip them with the knowledge and skills necessary to practice safely and sustainably in unsupervised settings. Thus, the development of specific postgraduate programmes has been urgently needed to provide pathways to vocational training and specialization in rural medicine appropriate to the Pacific region. Rocketship Pacific Ltd. (Rocketship) is an international health charity, based in Australia, dedicated to improving health in Pacific island countries through stronger primary care. Rocketship's particular focus to date has been on education and capacity-building for doctors and nurses working in rural communities and outer-island facilities. Since 2015, Rocketship has been working in partnership with the Ministries of Health and other key partners in Solomon Islands, Timor-Leste, Tonga and Vanuatu to design and deliver postgraduate training programmes in the core generalist disciplines family, community and rural hospital medicine. To date, this has resulted in new postgraduate Family Medicine courses being established in Timor-Leste and Tonga; a rural medical workforce support programme being delivered in Vanuatu; and a new Postgraduate Diploma in Rural Generalist Medicine being designed in Solomon Islands. These new programmes, as well as other notable initiatives elsewhere in the Pacific such as the Master of Medicine (Rural) programme in Papua New Guinea, the Diploma and Master of Family Medicine programme in Fiji and the Cook Islands Fellowship in General Practice, are transforming the health workforce in PICs with the potential to benefit island people across the “Blue Continent.” This paper describes the establishment of new postgraduate training programmes in family, community and rural hospital medicine in Timor-Leste, Tonga, Solomon Islands and Vanuatu from the perspective of Rocketship, the non-profit organization engaged by each country's Ministry of Health (or equivalent) to provide expert technical assistance with their initiative.

## Introduction

For decades, the populations of Pacific island countries (PICs) have suffered from a lack of access to health care. Like most developing countries in the world, the health workforce in PICs has for many years been too small, too specialized and too centralized, with the majority of clinicians—particularly doctors—concentrated in the urban centers (1). This is despite the fact that, in-keeping with the global trends, the majority of the populations of these islands live in rural areas and experience greater health burdens. In their first-ever regional meeting, held in Yanuca island in Fiji in 1995, the health ministers of PICs signed the “Yanuca Declaration,” committing to a “Healthy Islands” vision for the Pacific, making clear that strengthening primary healthcare (PHC) was the principle strategy to achieve that goal (2, 3). The following years witnessed growing alarm about the likely impacts of both non-communicable diseases (NCDs) and climate change in PICs, with these two major health threats being considered alongside communicable diseases as representing the “triple burden of disease” in the region (4). These concerns have unfortunately proven accurate, with PICs now occupying the majority of the top rankings of countries around the world with the highest burdens of NCDs (5) and PICs widely acknowledged as the “canaries in the coal-mine” with respect to the impacts of climate change, including on health (6).

The commitment to the “Healthy Islands” vision was reaffirmed 20 years later at the corresponding Pacific Health Ministers Meeting, in which it was acknowledged that the efforts and progress over the previous two decades had been relevant, but fell far short of achieving the goals of universal health access and improved health outcomes for Pacific island people (7). Despite the establishment and growth of nursing colleges, such as those in Vanuatu and Solomon Islands, the systems of training, deploying and supporting health professionals in PICs still lacked the capacity to produce sufficient numbers of clinicians and the vast majority of the region's doctors were concentrated in the referral hospitals. The region's main medical schools, Fiji National University (FNU, formerly known as the Fiji School of Medicine) and the University of Papua New Guinea (UPNG), require students from other PICs to relocate to Fiji and PNG for the duration of their undergraduate training, with only very small numbers of medical students from the other countries—typically one or two—graduating and returning home each year. While both institutions also provide postgraduate training, this requires several more years living abroad and these opportunities have, until recently, been limited to the traditional specialties of surgery, medicine, anesthetics, pediatrics and obstetrics and gynecology, with the more recent addition of emergency medicine. As a result, many outer-island hospitals and health centers across the Pacific region have had no permanent medical staff for many years, with those who have been deployed in the rural facilities typically having little, if any, postgraduate training.

In an attempt to address this rural medical workforce crisis, the governments of several PICs sought assistance from outside the region, with one potential solution clearly standing out: Cuba. Since the 1960s, Cuba has developed a reputation for having a health system that performs relatively well, with its citizens enjoying better access to healthcare and longer life expectancies than those of many developed countries, including—as is frequently and gleefully pointed out—the United States (8, 9). Cuba's deployment of hundreds of thousands of their doctors to work in over a hundred countries during that time has resulted in the “Cuban medical brigade” becoming the country's most valuable export (10, 11). Furthermore, the establishment of the Escuela Latinoamericana de Medicina (ELAM—the Latin American School of Medicine) has enabled around one hundred of those countries to send their own students to train as doctors in Cuba, usually on full scholarships. The ELAM programme produces thousands of medical graduates each year, who return home to their countries of origin—mostly developing countries—after spending 6–7 years living in Cuba, learning medicine in Spanish. In terms of its impact on global health, Cuba's system of “medical internationalism” has been nothing short of a revolution.

In the early 2,000 s, representatives of countries in the south and western Pacific region began negotiations with the Cuban government to host Cuban doctors as clinicians and trainers and send students to ELAM to become doctors. Timor-Leste was the first, sending nearly 700 students to ELAM in 2003–2004, followed by—in cohorts ranging in size from 3 to 25—Kiribati in 2007, Solomon Islands and Vanuatu in 2008, Tonga and Tuvalu in 2009 and Fiji in 2010 (12, 13).

As the 20th anniversary of the Yanuca Declaration approached and preparations began for the return of the first cohorts of ELAM graduates to their Pacific island homes, several of those governments requested assistance in developing programmes to integrate the new graduates. A particular focus, of course, was in placing the graduate doctors in the rural and outer-island facilities, in order to improve health access and outcomes for those communities. This was complicated by emerging reports of gaps in the knowledge and skills of the medical graduates trained in the ELAM system. At the same time, the renewal of the region's health ministers' commitment to the “Healthy Islands” vision led to a belated recognition of the dearth of postgraduate training pathways in what may be considered the primary care or generalist specialties—family, community and rural hospital medicine—and a renewed interest in developing such programmes in the Pacific region. While UPNG had established a Master of Medicine (Rural) programme, providing a narrow but robust pipeline of broadly-skilled rural medical specialists (14), and a small programme had been set up in Cook Islands to train doctors to diploma level as rural general practitioners, supported by the University of Otago and the Royal New Zealand College of General Practitioners (15), no other primary care or generalist medical training programmes existed in the Pacific.

Given the clear need for such postgraduate generalist training programmes to be established, and with strong support from health leaders in Fiji, Tuvalu and Vanuatu, in particular, the concept for a new and unique collaboration was born. The concept would draw on the previous two decades of rural medical workforce development experience in Australia and would link Ministries of Health and training institutions in the Pacific with, *inter alia*, the Australian College of Rural and Remote Medicine (ACRRM), the Remote Vocational Training Scheme (RVTS) and the Rural Generalist Pathway (RGP) in Queensland. Each of these initiatives was undertaken with the aim of improving health access and outcomes for rural communities, with the combined effect that Australia was, by the time of the inaugural World Summit on Rural Generalist Medicine in 2013, acknowledged as the undisputed world leader in the field (16). The outcomes statement from that summit, known as the “Cairns Consensus,” defined the concept of rural generalist medicine, affirmed its critical importance and outlined pathways by which doctors could train in the discipline.

The scope of rural generalist medicine, as defined in the Cairns Consensus, is as follows:

- Comprehensive primary care for individuals, families and communities;
- Hospital in-patient and/or related secondary medical care in the institutional, home or ambulatory setting;
- Emergency care;
- Extended and evolving service in one or more areas of focused cognitive and/or procedural practice as required to sustain needed health services locally among a network of colleagues;
- A population health approach that is relevant to the community;
- Working as part of a multi-professional and multi-disciplinary team of colleagues, both local and distant, to provide services within a “system of care” that is aligned and responsive to community needs.

The few Pacific island participants at the Summit took that message home and, with the support of several high-level representatives from ACRRM, RVTS and RGP, an international health non-government organization (NGO) was founded with a mission to assist PICs in improving the health of their communities through stronger primary care and a particular focus on the establishment and delivery of rural generalist training programmes. The new organization was named “Rocketship”—an acronym for Remote Opportunities for Clinical Knowledge, Education, Training and Support for Health In the Pacific.

This paper addresses a significant gap in the literature on human resources for health in low- and middle-income countries, including in the Pacific region, by presenting four case studies of rural medical workforce development over the last five years in Timor-Leste, Vanuatu, Tonga and Solomon Islands. The paper describes the innovative partnership arrangements that have led to each country establishing postgraduate training pathways in family, community and rural hospital medicine (noting that these terms are used more or less interchangeably in the Pacific region, even if a clearer distinction is usually made elsewhere). These programmes, while at different stages of development, have all been made possible with the support of Rocketship.

## Context

### Timor-Leste

As the first country in the region to engage with Cuban medical internationalism, and the one with by far the greatest number of graduates from the ELAM system, Timor-Leste is unique within this group of case studies. At the time of its independence in 2002, Timor-Leste had <50 doctors serving its population of ~1 million. By late 2012, it had close to one thousand doctors, with almost all of them either trained in Cuba or by Cuban doctors in Timor-Leste (17). The Universidade Nacional Timor Lorosa'e (UNTL—National University of Timor-Leste) established a medical school, with ELAM support, and hundreds of new graduates were sent to work in community health centers (CHCs) under the government's “Doctors For The Districts” initiative. Unfortunately, the experiment did not prove especially successful (13, 18). The experience of the first years of the programme led to a recognition of some critical shortfalls in the undergraduate training programmes in question and the need to provide further, formal postgraduate training to enable the new graduates to practise safely and competently across the scope of generalist clinical medicine to meet the health needs of their communities. Over the period 2013–2014, a plan for the country's first Family Medicine Programme (FMP) was developed, with the objective of providing Timorese doctors with a dedicated training pathway “to acquire the necessary knowledge, skills and behaviors to support their delivery of essential health services at the community health level” (19). The Royal Australasian College of Surgeons (RACS), with an office in the national referral hospital, funding from the Australian government and a strong track record of clinical service and postgraduate training support in Timor-Leste, requested Rocketship's assistance to design and implement the FMP for its first cohort in 2015.

### Vanuatu

In 2013, it was estimated that the health workforce providing care to Vanuatu's population of ~250,000 consisted of 46 doctors, 335 nurses and 62 midwives, along with 206 village health workers (VHWs) spread across aid posts in rural communities (20). This translated to a health worker to population ratio (excluding VHWs) of 1.77 per 1,000 population—one of the lowest in the Pacific region and well below the minimum threshold of 2.3–4.1 per 1,000 recommended by the World Health Organization (21). In 2008, as part of the implementation plan for Vanuatu's own “Healthy Islands” primary care systems strengthening strategy (22), the first cohort of students from Vanuatu were sent to Cuba to train as doctors at ELAM, with two subsequent cohorts making up a total of 37 by 2012 (12). The arrival of the first 14 of these graduates in 2015, therefore, represented a ~30% increase in the total number of doctors in the country. This increased to a roughly 50% increase over the next three years as the remaining graduates returned home. With almost all of the country's doctors concentrated in the two largest population centers, only a third practising as generalists in hospital and community primary care settings, and approximately one third of Vanuatu's hospitals having no doctors at all, there was a strong political imperative for the new graduates to be deployed to the outer-island facilities as quickly as possible. From 2015 to 2019, the Vanuatu Ministry of Health (MoH) requested support from the various managing contractors of Australian government funding for Vanuatu's health sector (including Health Specialists Limited and Aspen Medical) to redesign the national junior doctor training programme to integrate the new graduates and strengthen the medical capacity of the outer-island facilities. This, in turn, led to Rocketship's engagement to provide clinical service and training support in Vanuatu, with a focus on the new medical graduates and the chronically under-staffed rural hospitals—a partnership made possible due to Rocketship's previous collaborations with the Vanuatu MoH conducting professional development needs assessments for primary care nurses working in community settings, as well as delivering *ad-hoc* trainings for rural nurses and doctor-led clinics in remote villages.

### Tonga

Tonga was one of the last countries in the Pacific to send students to train as doctors in Cuba, and even those were very few in number, but it did so in acknowledgment of the fact that its population of ~100,000 lacked the resources to provide adequate medical care and manage the country's NCD crisis, in particular (23). With renewed commitment to growing and strengthening its health workforce in its national Strategic Development Framework (2015–2025) and Health Strategic Plan (2015–2020), the government of the Kingdom of Tonga began exploring innovative solutions to train Tongan doctors as specialist-level generalists. This initiative reflected the government's commitment to improving health access and outcomes in rural communities and outer islands, as per the MoH's Package of Essential Health Services (24). Tonga's high-level representative at the Third World Summit on Rural Generalist Medicine in Australia in 2017 thereby requested Rocketship's support to establish an in-country training programme for Tongan doctors, based on the model pioneered by Rocketship, RACS and the Timorese MoH with the FMP in Timor-Leste.

### Solomon Islands

The population of Solomon Islands is ~650,000 people spread across some 300 inhabited islands. The country's medical workforce, however, is highly concentrated in the capital city, with over 75% of the country's doctors based at the National Referral Hospital and large urban clinics in Honiara, where <15% of the population reside. In an attempt to address not only the maldistribution of the medical workforce, but the larger health burden borne by rural communities, the Solomon Islands Ministry of Health and Medical Services (MHMS) sent several dozen students to train as doctors in Cuba. When these graduates began to return, in relatively large numbers, in 2014, the substantial expansion of the country's medical workforce was initially viewed very positively, with the anticipated growth and professional development of the new graduates incorporated into long-term medical workforce planning on the part of the MHMS (25). However, when over the ensuing years similar gaps were identified in the graduates' knowledge and skills as had been demonstrated previously in Timor-Leste and concurrently in Kiribati and Vanuatu, it was acknowledged that a new, specific programme was required to train Solomon Islands doctors as rural medical specialists. The managing contractor for Australian government health sector funding, AVI (previously known as Australian Volunteers International), put out a competitive tender in 2019 to conduct a feasibility study of the establishment of a Postgraduate Diploma in Rural Medicine in Solomon Islands, which was ultimately awarded to Rocketship.

## Development of Programmes

The development of the abovementioned programmes took place quite differently in each country, although the overall objective of growing a medical workforce equipped with the knowledge and skills necessary to provide safe, quality care across the scope of rural generalist practice was essentially identical. What follows is a brief overview of the process, structure and outcomes of each country's establishment of such programmes with support from Rocketship and other partners.

### Timor-Leste

The FMP in Timor-Leste was designed as a two-year diploma programme, with the first year consisting of clinical rotations at the national referral hospital in Dili and the second year focused on training based in—and for practice specific to—the CHCs. The RACS-Rocketship partnership was established to enable delivery of the critically important community-based training phase. This followed a review of the initial FMP curriculum by members of the Rocketship team, at the request of RACS, which identified some critical gaps in the proposed educational and clinical apprenticeship programme in its second year, from the perspectives of core curriculum content, skills development and training support. The almost complete lack at the time of qualified family physicians in Timor-Leste available to mentor, supervise, support and act as role models for the FMP trainees was a particularly critical factor leading to the formation of this partnership.

From January to June 2016, a pilot programme was implemented that saw experienced rural generalist medical educators, recruited via Rocketship, coordinated by RACS and endorsed by the Timorese MoH, travel to Dili on a one-week-in-three rotating roster to deliver on-site supervision and teaching for the 12 trainees progressing through their second year of the FMP. This fly-in/fly-out (FIFO) model of clinical and education support was backed up by a Technical Advisory Group (TAG), comprised of the Rocketship Board of Directors—all leaders in the field of rural generalist medicine in Australia—who undertook a further, more detailed review of the FMP curriculum, drafted the teaching schedule for the visiting trainers, hosted regular team teleconferences and provided additional administrative support. The TAG also worked with the three-member Trainer Team to create the multiple choice question (MCQ) paper used in the final exams for the FMP trainees, along with a clinical case-based discussion assessment.

During each visit by the Trainer Team, the schedule of activities included workplace-based teaching, supervised clinical activities, discussion of “best practice” primary medical care and practical skills training. The topics covered over the 6-month period of collaboration were drawn from the educational programme proposed by Rocketship in relation to the curriculum review. The results of the final assessments were that eight of the first cohort of 12 FMP trainees passed both the abovementioned examinations to the standard of UNTL, as the awarding academic institution, thus successfully completing the inaugural programme. Rocketship went on to provide a scaled-back package of technical support for the final assessments of the FMP cohorts in 2017 and 2018. Over this period, in-country training capacity was built and responsibility for delivery of the community-based phase of the programme was transferred back to partners in Timor-Leste (notably RACS and the NGO Maluk Timor), as had always been the aim.

### Vanuatu

The Vanuatu MoH, in anticipation of the need for an intensive programme of support for the new medical graduates returning home from Cuba, created a role as Academic Programme Leader, which was funded by the Australian government, via the managing contractor in place in 2015, and taken up by one of the authors of this paper (LM). Over a period of several months and based on wide-ranging consultations, both with senior clinicians and policy-makers in Vanuatu and programme coordinators in other countries with similar programme (including Kiribati and Solomon Islands), a 3-month trainee internship programme was developed for Vanuatu to provide the necessary “bridging” for the ELAM graduates, with a revised and strengthened 2-year internship to follow.

The first cohort of 14 graduates arrived in late 2015. The majority successfully completed the trainee internship programme, as determined by the standards set by Vanuatu's national Medical Registration and Training Committee, and proceeded to internship in early 2016. Five were unsuccessful in their assessments and repeated the trainee internship programme for periods ranging from 3–9 months, with additional evaluations being conducted prior to their eventual transition to internship. During the trainee internship phase, Rocketship was requested by the Vanuatu MoH to provide short-term clinical cover for both the national referral hospital, which had no doctors working in the Emergency Department, as well as for the largest and busiest of the country's doctorless facilities—Lenakel Hospital in the southern province of Tanna. Three experienced rural generalists were therefore deployed by Rocketship to support the two facilities over a period of several months. In 2018, following completion of the 2-year internship programme which, for the first time in Vanuatu, included a specific curriculum in rural medicine (strongly influenced by Rocketship's experience and expertise), the junior doctors began extended placements in the outer-island hospitals and health centers. Over the following 18 months, Rocketship was once again engaged by the Australian government's managing contractor to provide additional supervisory and training support for the rural-based doctors. This led to Rocketship deploying 12 senior clinicians—predominantly rural generalists, with a small number of emergency physicians—to the country's two largest hospitals (Vila Central and Northern Provincial), and sending the most experienced of those to the outer-island facilities to provide feedback on and support to the junior doctors working, mostly alone and unsupervised, in those remote locations.

Despite significant enthusiasm on the part of several of Vanuatu's junior doctor cohort to specialize in rural medicine—as evidenced in their responses to surveys about their career ambitions when they first returned home from Cuba and following completion of their internship—no such pathway has yet been established in Vanuatu. In 2019, the first ni-Vanuatu doctor was enrolled in the UPNG Master of Medicine (Rural) programme, providing a small glimmer of hope for the longer-term future of rural medical workforce strengthening in Vanuatu.

### Tonga

A key enabler of the corresponding postgraduate training pathway in Tonga was the establishment of new Diploma and Master of Family Medicine programmes at FNU. This process involved Rocketship from an early stage, with representatives of the organization conducting a scoping visit to Fiji at the request of the FNU Dean of Medicine in 2014, conducting a series of consultations with both MoH and community representatives and strongly recommending that the university establish a postgraduate generalist training programme that would be open to trainees from across the Pacific region. This eventually transpired, with FNU launching a Diploma in Family Medicine programme in early 2018, with academic support from James Cook University in Australia. By this time, Rocketship's consultations and technical advice in Tonga had led to a partnership being created between Rocketship and the Tonga MoH, with funding from the Australian government to enable four Tongan trainees to commence the FNU Diploma, with the critical innovation being that they could remain living and working in Tonga for the duration of their training, rather than needing to relocate to Fiji. This was made possible due to a parallel partnership arrangement between Rocketship and FNU, whereby the university delegated key elements of the supervision, workplace-based training and assessments to Rocketship's Trainer Team—a new group of four experienced rural generalist medical educators co-led by one of the members of the previous Trainer Team that Rocketship had deployed in Timor-Leste. Using a similar FIFO model of regular country visits and remote mentorship and learning support, Rocketship's Trainer Team provided a 12-month education platform from which the four trainees were all able to successfully complete their assessments and be awarded their diplomas. Three of those achieved sufficiently high marks to be able to progress to the Master's programme, with a further four enrolling in the Diploma programme in 2020.

### Solomon Islands

The most recent of the four countries' training initiatives, the establishment of the Postgraduate Diploma in Rural Medicine (PGDRM) in Solomon Islands is, at the time of writing, yet to be officially approved by the MHMS. After Rocketship was awarded the contract to conduct the feasibility study of the proposed programme in late 2019, a series of consultations was held with representatives of the intended partner organizations—namely Solomon Islands National University (SINU) as the academic institution; DFAT as the funder; AVI as the managing contractor; and the MHMS, including the national medical training committee, the National Referral Hospital and the provincial hospitals intended to be used as training sites. The consultations were conducted over a period of 5 months, including two visits to Solomon Islands involving four Rocketship representatives. The findings of the study were that the proposed new programme was urgently needed and should be feasible, although there were significant hurdles that would have to be overcome. Among the most important of these obstacles are: (1) there are currently no rural medical specialists in Solomon Islands; (2) SINU, the proposed host academic institution, has neither a medical school nor any track record of training doctors; and (3) the internet connectivity across the country is poor at present, particularly in the outer islands. The curriculum that has been drafted is robust and appropriate, as determined by the TAG assembled by Rocketship to support the feasibility study—a group that included two Rocketship Directors, a former President and a former Director of ACRRM, the former Director of the Queensland Rural Generalist Pathway, a Professor of General Practice in Australia and the Director of the Papua New Guinea Master of Rural Medicine programme. There appears to be strong support for the PGDRM from policy-makers, clinicians and communities, as well as a commitment from the donor and managing contractor to fund the programme, at least in its initial phase(s). These latter factors, combined with Rocketship's capacity to partner with MHMS and SINU in the subsequent phases of design and delivery of the programme, would likely enable the initiative to progress. If it was to launch, and prove successful, it would become the first Diploma in Rural Medicine programme in the Pacific region (noting the UPNG programme is to Master's level, with no Diploma at present), which would, in turn, likely make it a very appealing model for other PICs to follow. However, the Covid-19 pandemic and the ensuing diversion of resources and restrictions on travel have hampered efforts to have the PGDRM ready to commence in early 2021.

## Findings and Implications

The aims and outcomes to date of these health workforce initiatives in PICs, focused as they are on improving healthcare access and health outcomes for rural communities, in particular by training specialists in family/community/rural hospital medicine, are closely aligned with the recommendations of the World Health Organization (WHO) in their seminal report, *Global strategy on human resources for health: workforce 2030* (26). Those same objectives are similarly aligned at the regional scale with the “Healthy Islands” vision for the Pacific (7). Some of these case studies from the Pacific have, in fact, already been used as exemplars in WHO guidelines for rural workforce development in low- and middle-income countries (27). The rural generalist model is acknowledged to be one of the most strategically valuable and effective means to achieve a higher standard of medical care for rural communities (28), including in island settings (16, 29), such that the establishment of these programmes is certainly timely, if not long overdue. Acknowledging the challenges of setting up such programmes in geographically isolated, resource-constrained environments—of which more below—the steps taken by the health leaders, senior clinicians and community representatives to build these pathways in each of these four countries is to be commended, as is the support from donors, particularly the Australian government, and regional partners such as FNU.

It is anticipated that these programmes will be expanded over the coming years, both in terms of other PICs joining the FNU Diploma and Master of Family Medicine programmes, likely adopting the in-country training model successfully trialed in Tonga with Rocketship support, and/or other PICs setting up their own programmes. Samoa is currently the only other PIC with its own university (the National University of Samoa) and medical school. The University of the South Pacific is a separate regional entity with campuses in most PICs, but no faculty of medicine at present.

The most valuable outcome of those programmes in the medium term should be the establishment and growth of a cohort of Master's-level (i.e., specialist) graduates in family, community and rural hospital medicine. With the right type and amount of professional development support, ideally aligned with WHO's guidance on health professionals' education and training (30), these Pacific island pioneer doctors have the potential to become supervisors, mentors and trainers of trainers, thus gradually eliminating, over time, the need for external and/or expatriate support.

Of particular interest, given the current global context, is the vital role of frontline primary care practitioners and the resilient nature of these training programmes that have been developed. Not only has this bold and innovative collaboration between Rocketship, the Tonga MoH and FNU proven the viability of in-country specialty training, thus enabling the trainees to remain in their own communities and the country to avoid losing valuable members of their medical workforce for postgraduate training overseas, the Family Medicine programme has proved to be the only specialty training programme at FNU to have been able to continue during the Covid-19 pandemic. This was made possible due to the distance education and mentorship model and provides an important precedent for future medical training initiatives in the Pacific region.

## Issues and Constraints

In addition to the aforementioned challenges related to distance, geography, poor IT infrastructure and resource constraints, it must be acknowledged that training programmes reliant on expatriate trainers providing remote support and relatively brief, infrequent visits are not an ideal, nor a long-term, solution. While the latter model is still preferable, in many respects, to trainees being required to relocate to a different country for the majority or duration of their training, the long-term viability and sustainability of these programmes will only be truly secured when sufficient doctors have graduated from the various pathways and been further trained as trainers, supervisors and mentors for future cohorts. To achieve this long-term goal, donors and regional partners, including Rocketship, must be willing to continue to support these initiatives over the coming years, until such a critical mass of qualified medical generalists in each country has been reached.

## Conclusion

The last 5 years have seen a seismic shift take place in the structure of the health workforce in several PICs, due to both the influx of new medical graduates, predominantly from Cuba, and the establishment of a variety of new postgraduate training programmes focused on the generalist disciplines of family, community and rural hospital medicine. Each of the four countries included here as case studies has demonstrated vision, leadership and foresight in capitalizing on the goodwill and expertise of Rocketship, other technical support organisations, regional academic institutions and the support of donors, particularly the Australian government, in enabling the new programmes to be designed and implemented in innovative ways, adapted to the context and requirements of each country. These initiatives are crucial in building a health workforce in the Pacific region that is of an adequate size, an appropriate distribution and a level of skill necessary to meet the needs of rural communities across the “Blue Continent.”

## Data Availability Statement

The raw data supporting the conclusions of this article will be made available by the authors, without undue reservation.

## Author Contributions

LM led the design and drafting of the manuscript. DM, SJ, and L'U all provided inputs, participated in the reviewing and revising processes and approved the final version of the manuscript. All authors contributed to the article and approved the submitted version.

## Conflict of Interest

LM, DM, and L'U currently, or have been SJ, unpaid volunteers for the international health non-profit organization Rocketship Pacific Ltd., including in their respective capacities as pro-bono directors on the Board. The authors declare that the research was conducted in the absence of any commercial or financial relationships that could be construed as a potential conflict of interest.
